# Left Bundle Branch Pacing: Current Knowledge and Future Prospects

**DOI:** 10.3389/fcvm.2021.630399

**Published:** 2021-03-23

**Authors:** Peng Liu, Qiaozhu Wang, Hongke Sun, Xinghua Qin, Qiangsun Zheng

**Affiliations:** ^1^Department of Cardiology, The Second Affiliate Hospital of Xi'an Jiaotong University, Xi'an, China; ^2^School of Life Sciences, Northwestern Polytechnical University, Xi'an, China

**Keywords:** left bundle branch pacing, physiological pacing, pacemaker, right ventricular apical pacing, cardiac resynchronization therapy

## Abstract

Cardiac pacing is an effective therapy for treating patients with bradycardia due to sinus node dysfunction or atrioventricular block. However, traditional right ventricular apical pacing (RVAP) causes electric and mechanical dyssynchrony, which is associated with increased risk for atrial arrhythmias and heart failure. Therefore, there is a need to develop a physiological pacing approach that activates the normal cardiac conduction and provides synchronized contraction of ventricles. Although His bundle pacing (HBP) has been widely used as a physiological pacing modality, it is limited by challenging implantation technique, unsatisfactory success rate in patients with wide QRS wave, high pacing capture threshold, and early battery depletion. Recently, the left bundle branch pacing (LBBP), defined as the capture of left bundle branch (LBB) via transventricular septal approach, has emerged as a newly physiological pacing modality. Results from early clinical studies have demonstrated LBBP's feasibility and safety, with rare complications and high success rate. Overall, this approach has been found to provide physiological pacing that guarantees electrical synchrony of the left ventricle with low pacing threshold. This was previously specifically characterized by narrow paced QRS duration, large R waves, fast synchronized left ventricular activation, and correction of left bundle branch block. Therefore, LBBP may be a potential alternative pacing modality for both RVAP and cardiac resynchronization therapy with HBP or biventricular pacing (BVP). However, the technique's widespread adaptation needs further validation to ascertain its safety and efficacy in randomized clinical trials. In this review, we discuss the current knowledge of LBBP.

## Introduction

Cardiac conduction disease is a serious health issue caused by the impairment to the integrity of conduction system. The molecular mechanisms of cardiac conduction disease have not been well-understood. To date, cardiac pacing is the only effective therapy for patients with symptomatic bradycardia. Traditional right ventricular apical pacing (RVAP) has been widely used for more than half a century, although the approach has been shown to cause electric and mechanical dyssynchrony, which exacerbates the risk of atrial fibrillation (AF), heart failure (HF), and even mortality (1–4). Moreover, other ventricular pacing sites, such as the right ventricular septal and right ventricular outflow tract, have been developed and applied to minimize the aforementioned potential adverse outcomes. However, their long-term outcomes have not been demonstrated to be superior to RVAP (5, 6). Cardiac resynchronization therapy (CRT), via biventricular pacing (BVP), is another pacing modality employed for treatment of HF. Clinical studies have demonstrated that CRT promotes left ventricular reverse remodeling, confers exercise tolerance, and reduces morbidity as well as mortality in patients with systolic HF (7). Although CRT's benefits are well demonstrated, the therapy has been associated with significantly high non-response rate (30–40%) (8). Furthermore, the BVP is a non-physiological approach that requires two leads to activate the ventricular myocardium and not the specialized conduction system.

Therefore, the physiological pacing technique that directly activates conduction systems becomes the focus of attention. Deshmukh et al. (10) first demonstrated feasibility of the permanent His bundle pacing (HBP) in patients with AF and dilated cardiomyopathy. Thereafter, multiple studies have confirmed the clinical benefits of permanent HBP (11, 12). Consequently, HBP has been recommended as a rescue modality for failed BVP and even a primary treatment for CRT (11, 13, 14). However, its clinical application in some patients has been limited by concerns associated with its technicalities, high pacing threshold, low R-wave amplitudes, and the potential to cause distal conduction block (12). Moreover, HBP cannot normalize the QRS duration in almost half of patients with left bundle branch block (LBBB) in the His Bundle Pacing vs. Coronary Sinus Pacing for Cardiac Resynchronization Therapy (His-SYNC) study (15). To address the above issues, researchers have recently developed the left bundle branch pacing (LBBP) therapy, as a novel pacing modality for delivering physiological pacing and ensure electrical synchrony of the left ventricle. Benefits of the LBBP technique, first reported by Huang et al. (16) in patients with dilated cardiomyopathy, have been demonstrated across several studies (17–21). Given the growing interest in pacing at the left bundle branch (LBB) region, we will summarize the current knowledge in LBBP, from anatomy to definition, implantation technique, complication, short-term clinical outcomes, potential advantages, and future directions, in order to provide comprehensive insights to help in understanding of this pacing modality.

## Anatomy of the LBB

**Figure 1:**
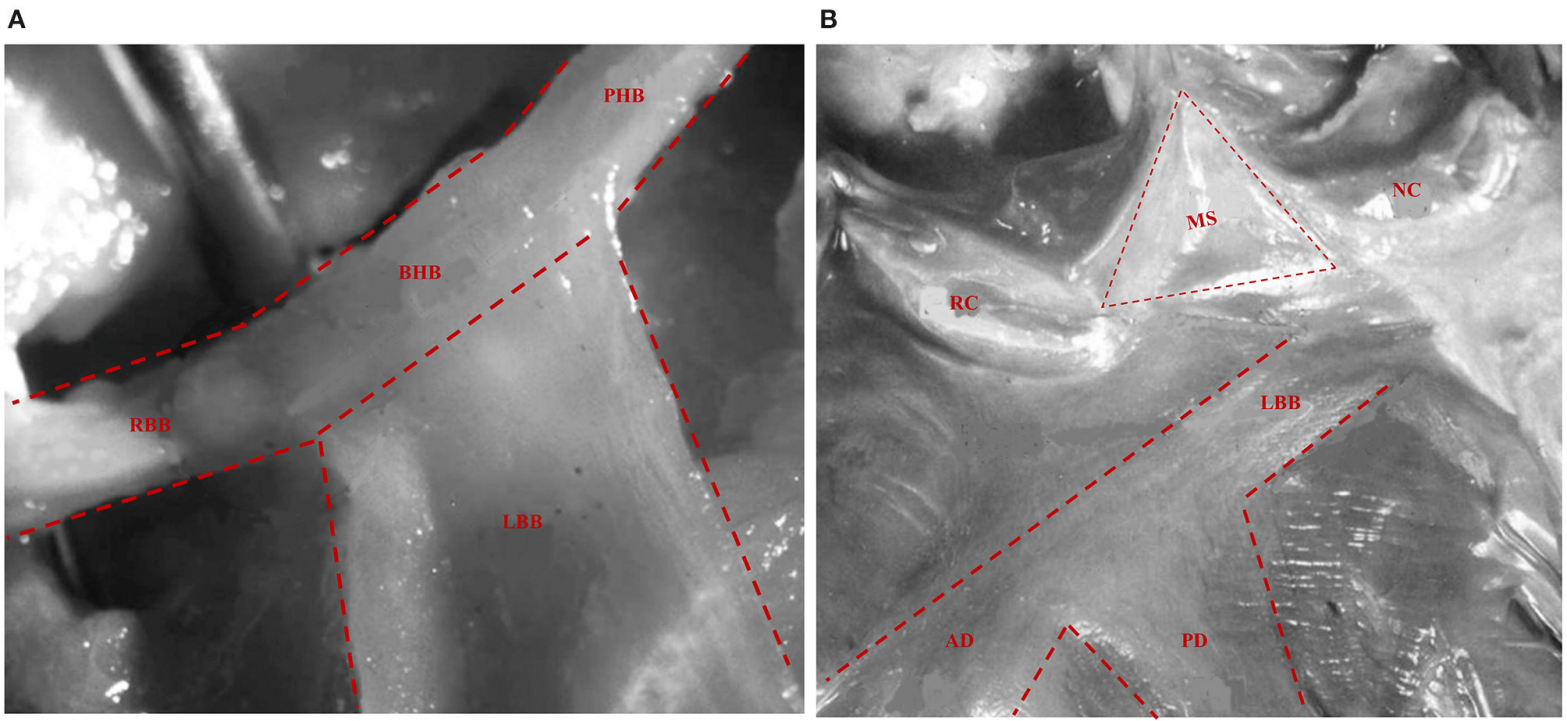
The anatomy of His bundle and LBB. **(A)** The His bundle comprises two segments: PHB and BHB. LBB originates from the BHB of His located below the MS. The RBB appears as a continuation of the bundle of His after the LBB has been given off. **(B)** LBB can be seen underlying the endocardium below the MS, which is encompassed between the NC and RC aortic cusps and the summit of the ventricular septum. Then, it produces its two main divisions, AD and PD, both heading the anterior and posterior papillary muscles, respectively (23). LBB, left bundle branch; PHB, penetrating portion of His bundle; BHB, branching portion of His bundle; MS, membranous septum; RBB, right bundle branch; NC, non-coronary aortic cusps; RC, right coronary aortic cusps; AD, anterior division; PD, posterior division.

**Figure 2:**
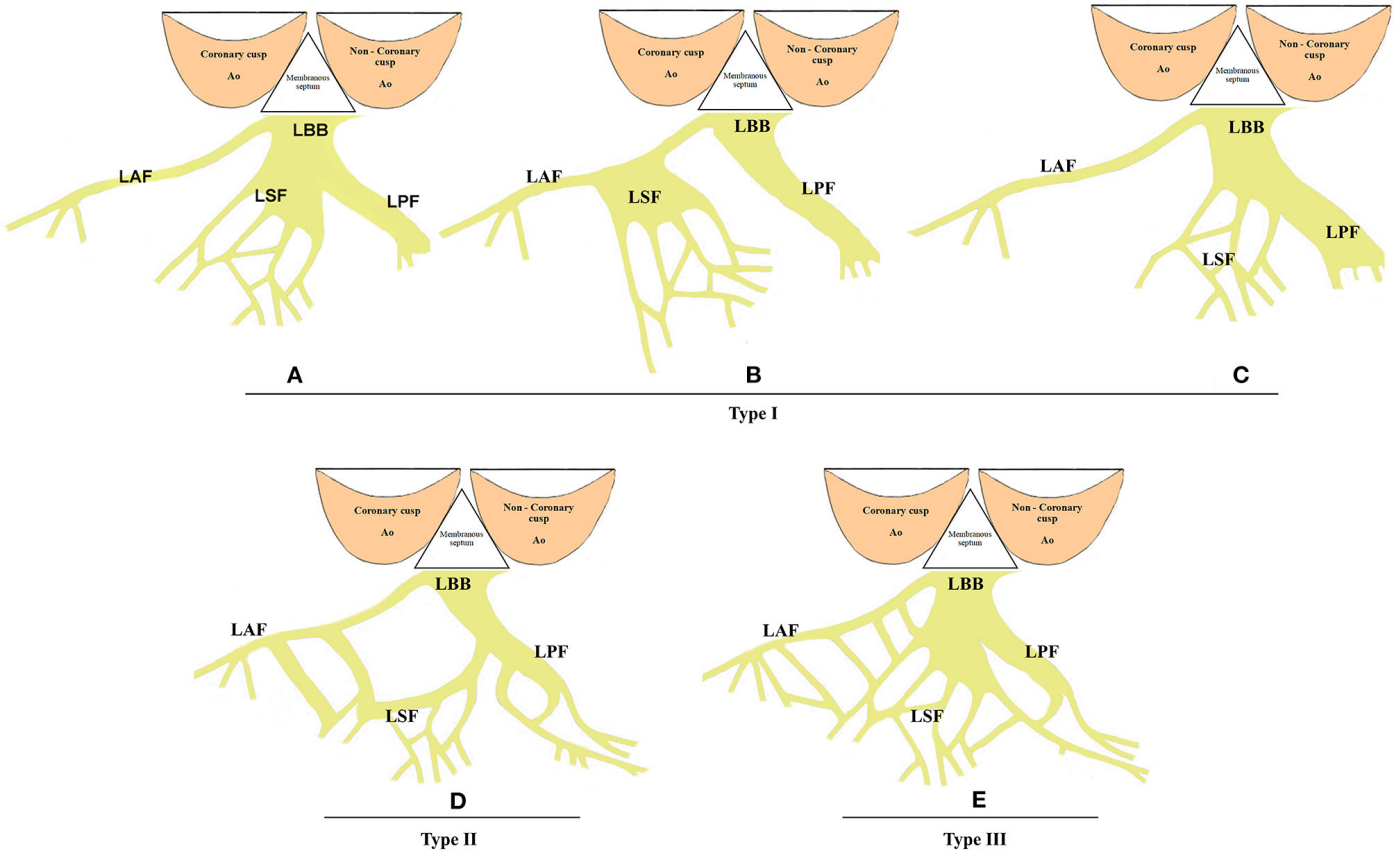
The anatomical variants of LSF. **(A–C)** The most common LSF pattern, known as type I. In this type, LSF may originate from the main LBB or any of its division (PD or AD). **(D)** Type II anatomical variants of LSF. The LSF branches concomitantly from the AD and PD. **(E)** In type III, LSF is a “fan-like interconnecting network.” LSF, left septal fascicle; LBB, left bundle branch; AD, anterior division; PD, posterior division.

The His bundle and its branches were first described by Tawara in 1906 (22). The bundle of His, a thin cylindrical fascicle that connects the atrioventricular node with bundle branches, has two segments, namely, the penetrating portion (PHB) and the branching portion (BHB). LBB originates from the BHB of His, located below the membranous septum (MS) (Figure 1A). All the fibers forming the LBB lie on the same plane giving a ribbon-like appearance beneath the endocardium of the subaortic septal region below the pars membranacea at the angle formed by the non-coronary and right coronary aortic cusps (23). The LBB's initial section is the narrowest, reaching its maximal width after extending about 10–15 mm (23). Its composition and distribution considerably vary across individuals. In some cases, two main divisions, anterior and posterior, that both head the anterior and posterior papillary muscles, respectively, appear soon after the origin of LBB (Figure 1B). Generally, the posterior division (PD) is thicker and shorter than the anterior division (AD) (24, 25), and in some cases, there are also well-defined left septal fascicle (LSF), which can arise from the PD and less frequently from the AD. Demoulin and Kulbertus (26) described the LSF's anatomical variants in 20 normal human hearts, with the most common pattern, which they called type I, showing a definite septal division. In this type, the LSF may originate from the main LBB or its division (PD or AD). In type II, the LSF branches concomitantly from AD and PD, whereas in type III, it appears as a “fan-like interconnecting network” (Figure 2) (11, 26–28). The existence and importance of LSF cannot be ignored. Particularly, these fibers are known to produce a network of interwoven strands that cover the inferior third of the septum, which avoids the widening of QRS when one of the divisions of the LBB is blocked (26, 28, 29). Overall, LBB's anatomical characteristics determine the feasibility of LBBP as a potential physiological pacing modality.

## LBBP Definition

**Figure 3:**
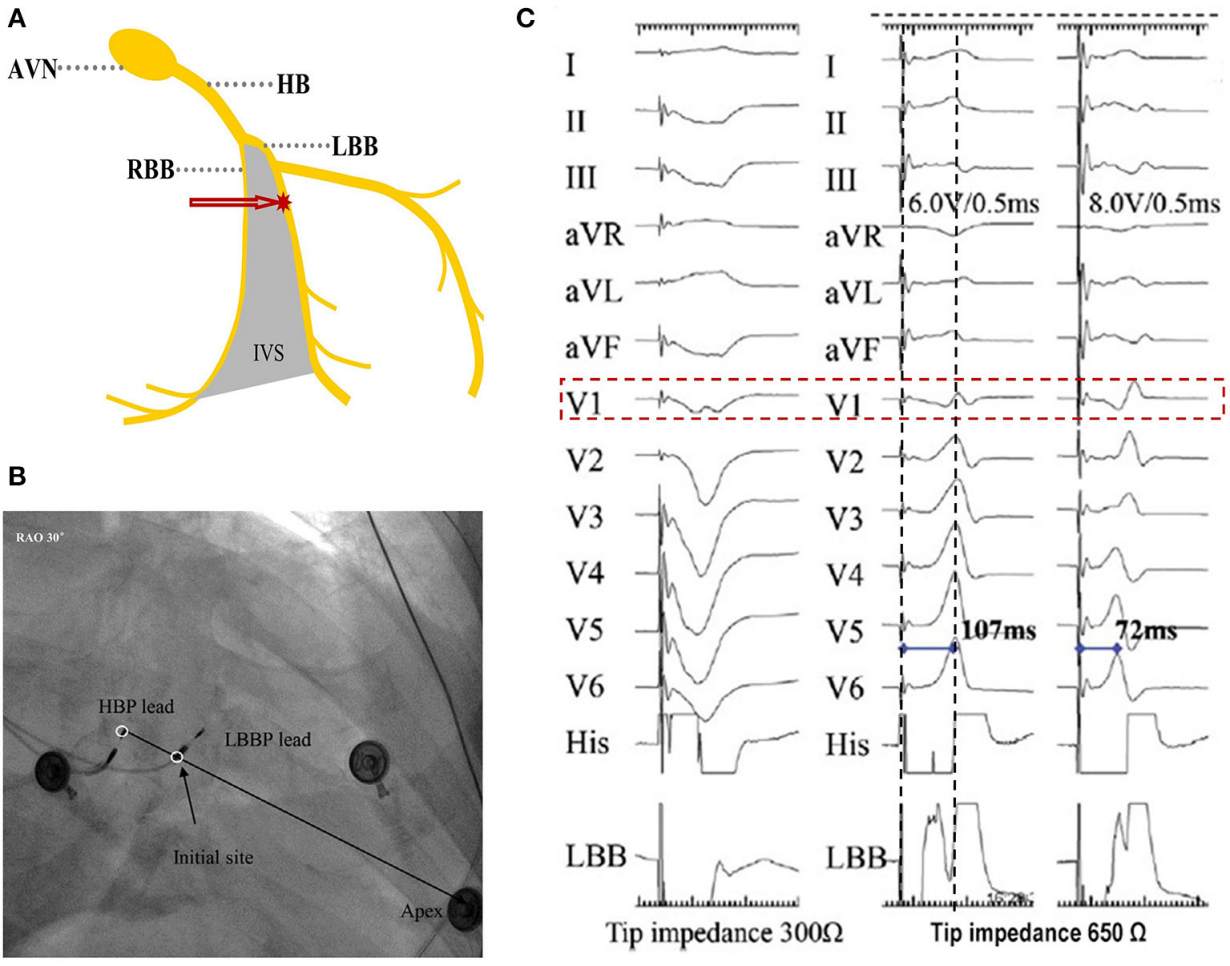
How to locate the site for LBBP and electrogram characteristics. **(A)** A photographic representation of LBBP. **(B)** Location of the HBP lead and LBBP leads in the right anterior oblique 30° view. **(C)** Paced morphology of “W” pattern with a notch at the nadir of the QRS in lead V1 and impedance of 300 Ω by unipolar tip pacing before fixation (left). Screwing the lead ~6–8 mm deep, the notch in lead V1 moved up and toward the end of the QRS with impedance of 650 Ω. Increased output, from 6.0 V/0.5 ms (middle) to 8.0 V/0.5 ms (right), caused the paced morphology to change to RBBB and the pLVAT to be shortened from 107 to 72 ms (9). HBP, His-bundle pacing; HB, His bundle; LBB, left bundle branch; LBBP, left bundle branch pacing; RBBB, right bundle branch block; pLVAT, peak left ventricular activation time; AVN, atrioventricular node; RBB, right bundle branch; IVS, interventricular septum.

LBBP is defined as capture of the LBB, usually with septal myocardium capture at low output (<1.0 V at 0.4 ms pulse width) (9, 30). In LBBP, the ventricular pacing lead is placed deep inside the interventricular septum 10–15 mm apical and ventricular to the distal His bundle region in the vicinity of the left bundle or its branches (Figures 3A,B) (9). The capture of LBB can be confirmed by some criteria described below, such as paced QRS morphology, peak left ventricular activation time (pLVAT), LBB potential, retrograde His or anterograde distal LBB potentials, programmed stimulation, and selective or non-selective LBBP.

### Paced QRS Morphology

The paced QRS morphology, during unipolar LBBP, shows the pattern of right bundle branch block (RBBB) in V1 lead (qR or Qr) or improving the LBB conduction in patients with LBBB (Figure 3C) (31, 32). The RBBB pattern is usually incomplete and is influenced by the level of capture of the distal His bundle or proximal left bundle, distal conduction system disease, and septal-Purkinje connections. However, the QRS morphology alone is not a good predictor of left bundle capture, because RBBB pattern may not be observed if the pacing site is located in the superior septum or near the distal His bundle or proximal left bundle (33). Furthermore, the left ventricle septal pacing (LVSP) without capturing the left bundle can also produce an RBBB pattern. The difference is that the LVSP has prolonged left ventricle (LV) free-wall activation compared with LBBP.

### pLVAT

The pLVAT is measured from the onset of the pacing spike to the peak of the R wave in the lead V5–6 (9). pLVAT is an indicator of the rapidity of LV free-wall activation used to identify the depth of pacing lead and capture of the LBB. Upon left bundle capture, pLVAT always remains short (<80 ms) and stable across different pacing outputs (Figure 3C). An increase in pLVAT, from high (10 V) to low (2 V) output, indicates the lead is away from the left bundle region and hence has to be carefully advanced slightly further to reach the left bundle. The current experience suggests a pLVAT <80 ms indicates LBB capture (9). However, pLVAT can be influenced by intraventricular conduction defects and ischemic cardiomyopathy with significant scar, necessitating further refinement of pLVAT's cut-off point.

### LBB Potential

**Figure 4:**
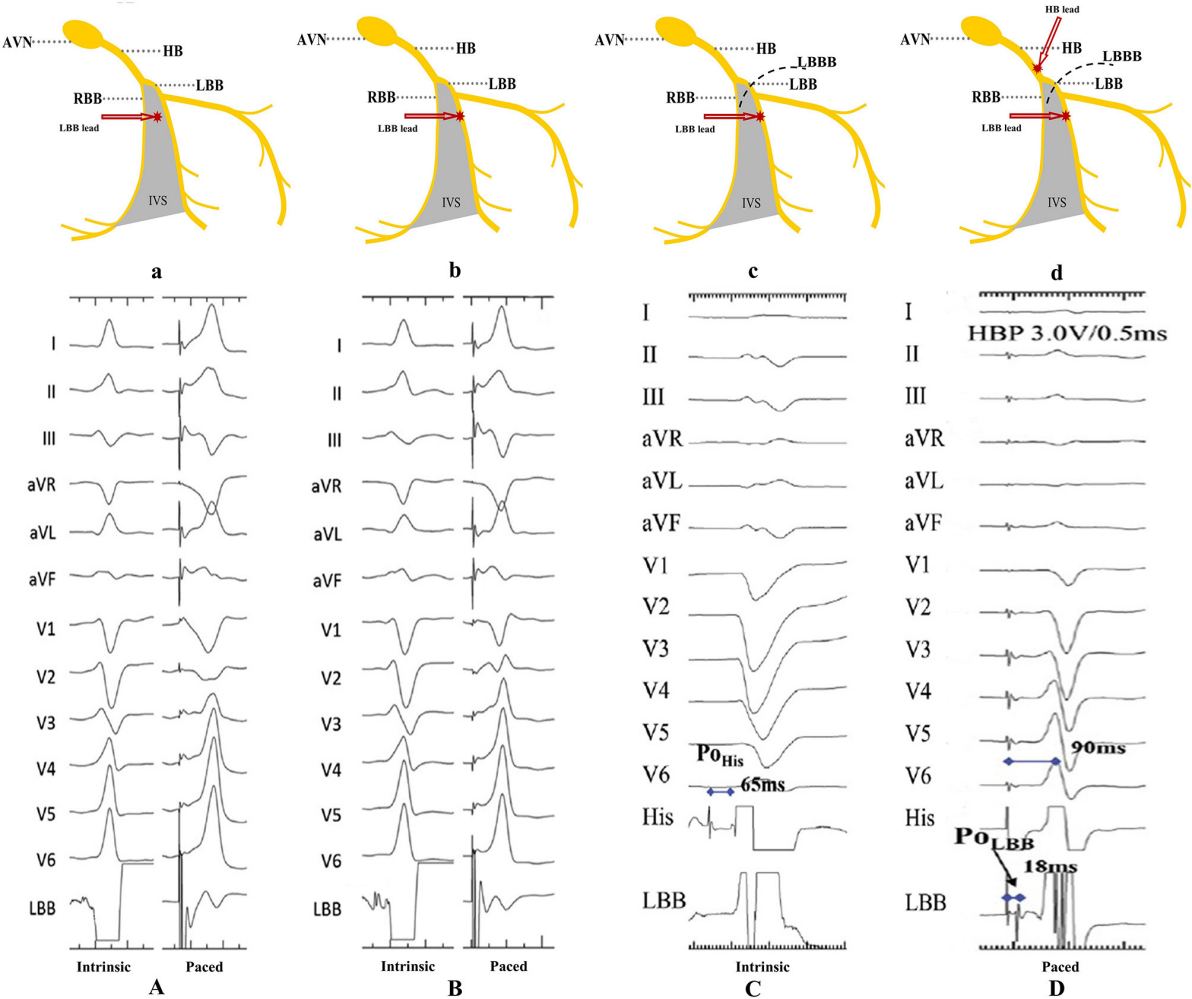
Recording of the LBB potential. **(A)** The LBB potential can be recorded when the pacing lead helix is approaching the LBB. **(B)** The LBB potential becomes larger when the lead is closer to or at the LBB. **(C,D)** The LBB potential cannot be recorded in patients with LBBB, unless LBB is corrected by HBP (9). AVN, atrioventricular node; HB, His bundle; LBB, left bundle branch; LBBB, left bundle branch block; RBB, right bundle branch; IVS, interventricular septum; Po_His_, His potential; Po_LBB_, left bundle branch potential.

LBB potential should always be recorded in patients without complete heart block (CHB) or complete LBBB. It is a sharp high-frequency deflection distance 15–30 ms to the onset of surface QRS (His potential to the ECG QRS onset is about 50 ms) (Figures 4A,B) (30, 34). LBB potential can help confirm lead depth and the level of conduction block. Interestingly, LBB potential can also be recorded in patients with LBBB, although it is limited to LBB conduction restoration via the HBP technique (Figures 4C,D) (35).

### Retrograde His or Anterograde Distal LBB Potentials

**Figure 5:**
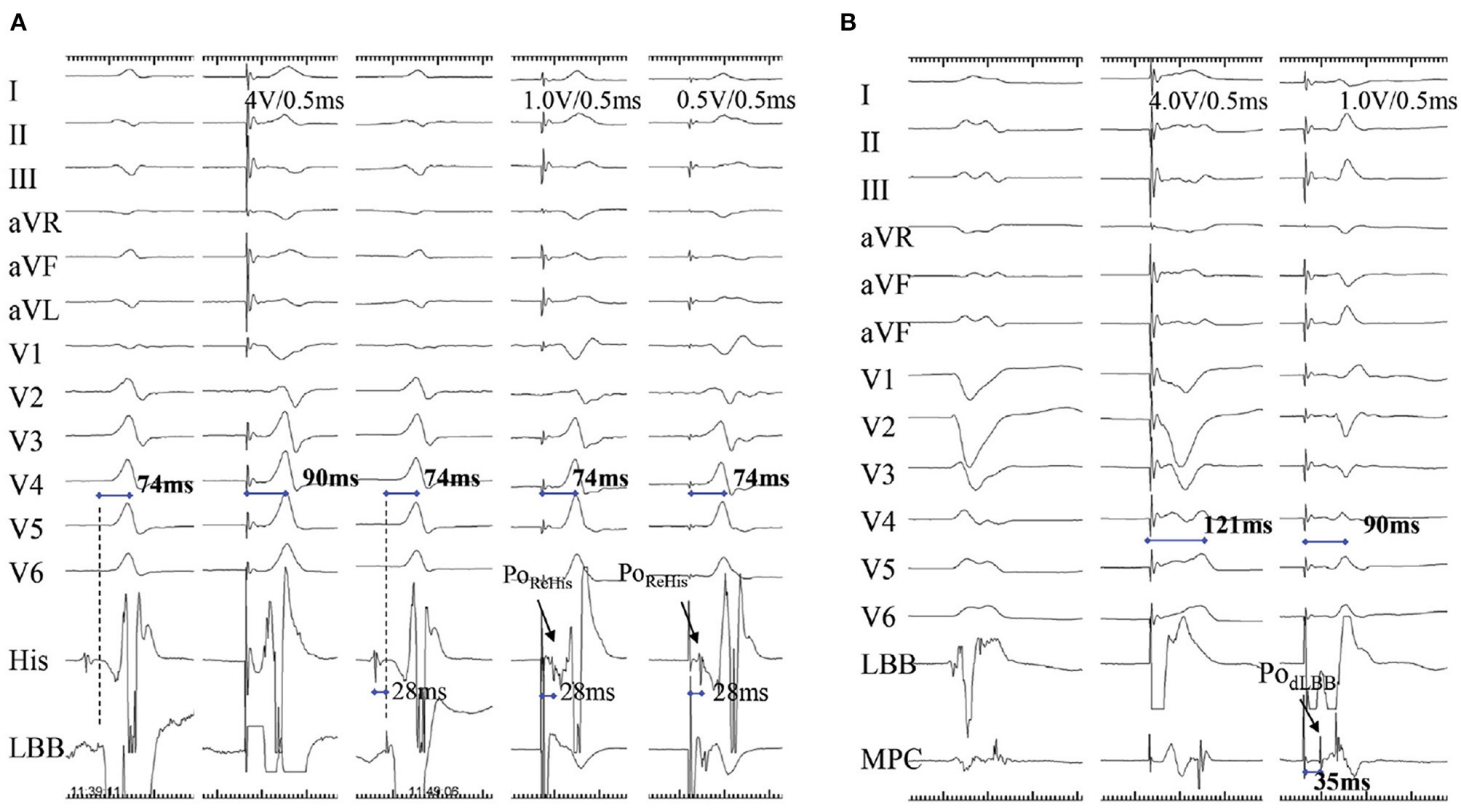
A demonstration of direct LBB capture using retrograde His potential and anterograde distal left conduction system potential. **(A)** A case with narrow QRS wave. There is a small Po_LBB_ and long pLVAT (90 ms) with septal pacing (first two beats). When the lead is advanced at a deeper site of septum, the non-selective and selective LBBP (last three beats) are visible, with larger Po_LBB_, shorter and constant pLVAT (74 ms), and Po_Rehis_ with stimulus to Po_Rehis_ interval of 28 ms at low and high outputs. **(B)** The Po_dLBB_ can be recorded after the ventricular EGM with MCP distal to the LBBP lead (first beat). The second beat shows the Po_dLBB_ remains after the ventricular EGM with 121 ms pLVAT during septal pacing, whereas the last indicates achievement of selective LBBP at a deeper site, Po_dLBB_ recorded ahead of ventricular EGM with shorter pLVAT (90 ms) (9). LBB, left bundle branch; pLVAT, peak left ventricular activation time; LBBP, left bundle branch pacing; MCP, multipolar placed; EGM, electrogram; Po_dLBB_, distal left bundle branch potential; Po_Rehis_, retrograde His potential.

Reverse His potential can be recorded during low-output LBBP, via direct capture of LBB, in patients without conduction disease (Figure 5A). Alternatively, stimulus to atrial intervals can be assessed during unipolar pacing from the LBBP lead tip (cathode at the LBB) and unipolar ring (anode at right ventricular septum). Here, the stimulus to atrial intervals would be markedly shorter than right ventricular septal pacing (RVSP) (36). Moreover, the anterograde distal LBB potential can also be considered as an indicator of LBB capture and can be recorded by multipolar catheter placed distal to the LBBP lead (Figure 5B) (9).

### Programmed Stimulation

In some cases, the abovementioned criteria may not be observed during lead implantation. Thus, programmed stimulation may be adopted, as an alternative method, for differentiating septal and LBB capture. For example, Jastrzebski et al. (37) demonstrated that programmed deep septal stimulation with a 600-ms basic drive train could identify 79.7% LBB capture in patients. Their results further showed that the average septal-myocardial refractory period was shorter than the LBB refractory period (263.0 ± 34.4 vs. 318.0 ± 37.4) (37). However, this approach is not applicable to patients with LBBB (37).

### Selective or Non-selective LBBP

**Figure 6:**
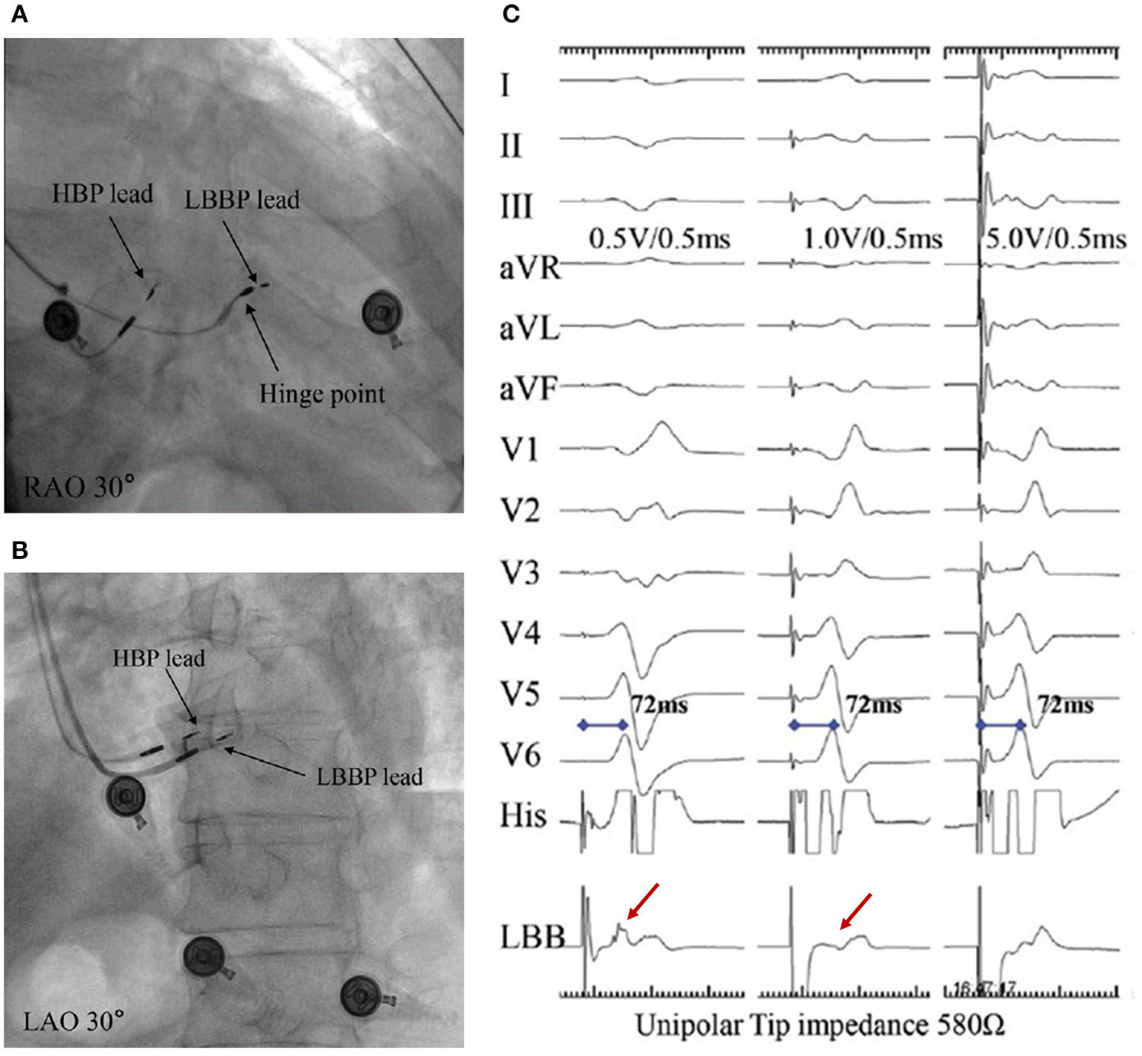
A demonstration of selective and non-selective LBBP and the depth of its lead in the septum. **(A)** Fulcrum sign. **(B)** Sheath angiography in the LAO 30° view demonstrating the depth of the LBBP lead inside the septum. **(C)** The presence of a discrete local EGM with short pLVAT (72 ms) during selective LBBP (first beat). The last two beats indicate a constant pLVAT at different pacing outputs without discrete component, which is considered as non-selective LBBP (9). LBBP, left bundle branch pacing; LAO, left anterior oblique; RAO, right anterior oblique; EGM, electrogram; pLVAT, peak left ventricular activation time.

LBBP can either be selective or non-selective, in a similar fashion to HBP. Selective LBBP captures only the LBB as a direct LBB capture sign. In fact, capturing both LBB and the adjacent local septal myocardium causes non-selective LBBP. While selective LBBP guarantees an isoelectric interval, between the pacing spike and the onset of surface QRS, this is not the case in non-selective LBBP (Figure 6C) (31). Moreover, a discrete local ventricular electrogram (EGM), separate from the pacing artifact, can only be seen on the LBBP lead at low pacing output (Figure 6C) (35). Apart from the aforementioned indicators, pLVAT duration in non-selective LBBP may be prolonged when the output changes from high to low (32). Moreover, there are also longer stimulus–His interval and stimulus-to-right atrial interval, compared to the selective LBBP (32, 34). However, Chen et al. (35) found that both selective and non-selective LBBP resulted in constant pLVAT at different pacing outputs, implying that pLVAT may be not a powerful indicator of selective or non-selective LBBP.

## LBBP Implantation

Evaluating the structure of the heart, especially the thickness of the basal interventricular septum and the presence of septal scars, is a crucial requirement before surgery. The SelectSecure lead (model 3830, Medtronic Inc., Minneapolis, USA) and Select Site C315 His or C304 His sheaths (Medtronic Inc., Minneapolis, USA) are used in operation, while an electrophysiological multichannel recorder is used to simultaneously document intracardiac EGMs and 12-lead ECG. Moreover, the Pacing System Analyzer (PSA) is used to test the pacing parameters and record intracardiac EGMs via the pacing lead. Generally, the operation process can be summarized as follows: (1) establishment of the venous access and determination of the initial LBBP site; (2) introducing a pacing lead into the right ventricle and screwing it into the interventricular septum (IVS) until the left ventricular septum is reached in the LBB areas; (3) assessing the lead depth into ventricular septum and confirming LBB capture; (4) removing the sheath and providing the slack; and (5) programming the pulse generator.

### The Initial Site for LBBP

In LBBP, the His bundle region or tricuspid valve annulus can be used as anatomic markers for the pacing site. The target site is about 10–15 mm below the His bundle region, based on an imaginary line drawn from the distal extent of the His bundle to the RV apex in right anterior oblique (RAO) 30° fluoroscopic view (Figure 3B). The use of fluoroscopic landmarks, such as quadripolar catheter or another 3830 lead, to locate LBBP's initial site is possibly helpful for beginners, while it is not a general recommendation. Pace mapping, at this site, will often show a “W” pattern in lead V1 with a notch at the nadir of the QS complex, a positive QRS in lead II, and biphasic QRS in lead III (Figure 3C) (31). It should be noted that the “W” pattern in lead V1 may not appear in ~20% of patients (38).

### Fixing the Lead

Once the site is confirmed, the C315 sheath (Medtronic Inc.) is rotated counterclockwise to maintain orientation of the lead tip perpendicular to the septal surface, thereby providing adequate support to allow screwing of the lead into the septum. Rapid lead rotations, three–four turns at a time by one or both hands, are suggested to achieve penetration of the lead body behind the screw into the septum. Thereafter, the lead is released and the rapid rotations are repeated. Advancing the lead deep inside the septum is expected to reveal the following parameters; (1) the notch on the nadir of “W” in lead V1 will gradually ascend up to form an R wave (Figure 3C); (2) unipolar pacing impedance gradually increases, before dropping by 100–200 Ω as the lead reaches the LV subendocardium; and (3) the left bundle branch injury current is present in 70% of the cases (20).

### Determining the Optimal Position of the Lead

The ultimate ideal lead location depends on parameters of pLVAT, unipolar pacing lead impedance, and the presence of LBB potential. Generally, the paced pLVAT duration measured in lead V5 or V6 is short and constant (<80 ms) at differential pacing output, whereas the paced QRS morphology indicates qR or rSR in lead V1 (Figure 3C). Moreover, LBB potential is an important marker in patients with narrow complex or RBBB at baseline (Figures 4A,B). Interestingly, premature complexes of RBBB pattern can appear during lead fixation, suggesting the presence of Purkinje potentials in patients with complete heart block or LBBB (38). The unipolar pacing impedance is preferably >500 Ω. Further rotations need to be avoided if pLVAT is short and constant (<80 ms), LBB potential recorded, or unipolar pacing impedance of around 500–550 Ω with low capture threshold (<1.0 V at 0.5 ms pulse width). The septum's lead depth is ~1.4 ± 0.23 cm (32). However, in cases where the LV is perforated, simply withdrawing the lead is not adequate and must be repositioned at a different location. During the procedure, lead depth can be determined by contrast injection and echocardiography (Figure 6B), whereas 3D mapping system can also be used to assess the depth of lead after lead fixation. Therefore, 3D mapping may be a valuable tool for LBBP if it could monitor lead depth in real-time (39).

### Removing the Sheath and Providing the Slack

When the lead is fixed, the sheath is pulled back into the right atrium, and the lead gently advanced to provide adequate slack. Improper and excessive slacks can cause lead dislodgement and late perforation, respectively. Furthermore, the pacing parameters need to be checked in both unipolar and bipolar modes, prior to slitting the sheath. The pacing lead can easily format an alpha loop after slitting the sheath, which can then be redressed by slowly retracting the lead, by applying a slight counterclockwise torque in RAO view (38).

### Programming the Pulse Generator

Unipolar, bipolar, and anodal capture thresholds need to be recorded, prior to programming the pulse generator. Additionally, atrioventricular (AV) delay programming should be individualized based on native AV conduction and bundle branch block, while the automatic AV search function is routinely turned on in patients with sinus node dysfunction and intermittent AVB. The RBB conduction delay, caused by LBB capture, can also be partly compensated via two means (38): (1) programming the output above the anodal threshold, as the anode captures the septum's right side, and (2) optimizing the AV delay to allow native fusion through RBB. However, the programming above anodal capture is optional when battery life is considered.

### Failure of LBBP

LBBP guarantees a high success rate, between 80 and 97% (18, 19, 32). LBBP's failure to advance the lead in the septum has been attributed to the difficulty in lead fixation, as well as other factors, including septal scar/fibrosis at the fixation site, tissue lodging into the helix, deformed sheath or helix, and, most commonly, inadequate sheath support or incorrect sheath orientation. In these cases, removing the tissue from the helix, using a 22–24 G needle, replacing the sheath or lead, and distally and inferiorly repositioning the lead may be helpful (9).

## LBBP-Associated Complications

### Septal Perforation and Thromboembolism

Septal perforation and thromboembolism represent the most common complications associated with LBBP. Specifically, septal perforation comprises acute and late lead perforation, with the acute condition reported in 3% of patients following LBBP implantation (32). Acute lead perforation into the LV cavity can be discerned by the diminution of R wave amplitude, increase in capture threshold, or an immediate fall in unipolar impedance below 500 Ω. To avoid perforation, it is important to evaluate the thickness of the basal interventricular septum and lead length (the lead helix is 1.8 mm long and is 9 mm away from the anode tip) (Figure 6A). Moreover, a contrast injection can be used to assess lead depth during operation in the left anterior oblique (LAO) 30° (Figure 6B). In cases where acute septal perforation occurs, the lead needs to be re-implanted at a different site. Although late septal perforation is rare, it is a potential LBBP complication. To date, only a single case of late septal perforation, which has similar characteristics to acute septal perforation, has been reported during follow-up (40). In addition, exposure of the helix to the LV cavity is thought to be a theoretical risk of thromboembolism, although this has not been experimentally proven. Thus, there is a need to carefully monitor patients during follow-up.

### RBB and Septal Arterial Injury

The RBB may be injured due to manipulation of the sheath at the basal septum below the His bundle region. Notably, ventricular backup pacing is recommended prior to LBBP lead implantation in patients with LBBB, because RBB injury may cause the AV to be completely blocked during the procedure. Moreover, injury to the coronary artery may also occur when the lead is placed deep in the proximal septum (41). To minimize this complication, clinicians are encouraged to place the lead at least 10 mm below the His bundle region.

### Lead Dislodgement

The risk of lead dislodgement is slightly higher than HBP. Previous studies have reported acute lead dislodgement in LBBP, with Vijayaraman et al. (32) demonstrating its occurrence in three out of 97 patients who underwent LBBP. To minimize the risk of dislodgement, it is imperative to ensure appropriate slack and satisfactory pacing parameters are put in place. Furthermore, follow-up is encouraged to confirm the risk of late lead prominence.

## Short-Term Clinical Outcomes of LBBP

### Early Case Reports That Employed LBBP

Although research on LBBP is still at the exploratory phase, results from recent clinical explorations have been encouraging. For example, Huang et al. (16) were the first group to report LBBB and dilated cardiomyopathy in a 72-year-old HF woman treated with LBBP. Specifically, they used a low pacing output to correct the LBBB with accompanying RBBB on the electrocardiogram. At 1-year follow-up, they found a 62% increase in the left ventricular ejection fraction (LVEF), from a baseline 32%. Moreover, the left ventricular end-diastolic diameter (LVEDD) had decreased from 76 to 42 mm, whereas the New York Heart Association (NYHA) class had improved from a baseline IV to I (16). Similarly, Li et al. (42) reported a patient who accepted LBBP because of symptomatic systolic HF and complete LBBB. LBBB was corrected (QRS duration <120 ms) by a capture threshold 0.5 V, with the authors observing a significant improvement in exercise tolerance, reduction in ventricular size, and recovery of left bundle branch conduction after 1 year of LBBP therapy (42). Furthermore, Wu et al. (43) reported the use of LBBP on a 74-years-old patient, with a LVEF and LVEDD of 34% and 62 mm, respectively, because of the RVAP-induced cardiomyopathy. They found that the patient's LVEF had increased to 63%, his LVEDD had decreased to 46 mm, and NYHA class had improved from III to I, after 6 months of LBBP. Moreover, they recorded a LBB capture threshold and R-wave amplitude of 0.5 V/0.5 ms and 20 mV, respectively (43). Vijayaraman and Panikkath (44) reported the successful application of LBBP in a patient who underwent bioprosthetic tricuspid valve replacement and whose proximal His bundle in the right atrium could not be located.

### Comparison of Short-Term Clinical Outcomes of LBBP and RVAP

Several prospective studies have demonstrated that permanent LBBP guarantees a stable threshold, a narrow QRS duration, and preserved left ventricular synchrony, with only a few complications (18–21, 32, 34, 35, 37, 45). For example, Hasumi et al. (46) attempted to implant LBBP in 21 patients with HBP failure in atrioventricular block and obtained a success rate of 81% (17/21). Particularly, the mean procedure time of LBBP implantation was <15 min, whereas the QRS duration was reduced from 116 ± 8.3 ms to 108 ± 4.2 ms. Moreover, the group achieved a significant narrowing of the QRS duration in four patients with LBBB (from 151 ± 4.0 to 122 ± 6.7 ms, *P* = 0.01), with a mean capture and LBBB correction thresholds of 0.77 ± 0.07 V/0.4 and 0.89 ± 0.14 V/0.4 ms, respectively. The speckle tracking echocardiogram revealed no significant deterioration in the left ventricular total longitudinal strain, relative to intrinsic rhythm, during LBBP. Moreover, the researchers observed no complications during the 6-month follow-up (46). On the other hand, Li et al. (19) evaluated the LBBP in 87 patients with sinus node dysfunction and atrioventricular conduction disease and achieved an 80.5% LBBP implantation success rate, with an average procedure time of 18.0 ± 8.8 min. Notably, the LBBP's QRS duration was significantly narrower than RVAP (113.2 ± 9.9 ms vs. 144.4 ± 12.8 ms, *P* < 0.001), whereas the pacing threshold was low and stable (0.76 ± 0.22 V). Moreover, the researchers observed no adverse events during 3-month follow-up (19). Vijayaraman et al. (32) recorded 93 (93/100) and 88% (21/24) LBBP implantation success rates in bradycardia and LBBB patients, respectively. From their findings, it was evident that LBBP could significantly lower QRS duration in patients with LBBB (137 ± 19 ms vs. 162 ± 21 ms, *P* < 0.001). Notably, the authors reported that three patients had acute lead dislodgments within 24 h, three others had ventricular septal lead perforation, whereas one developed pericardial effusion. However, they did not observe transient ischemic attacks or thromboembolism in any of the patients during the short-term follow-up (32). Chen et al. (18) compared ECG parameters between LBBP and RVAP and found significantly narrower QRS duration in LBBP than RVAP (111.85 ± 10.77 ms vs. 160.15 ± 15.04 ms, *P* < 0.001). Two patients, with LBBB correction by LBBP, exhibited reduced QRS durations, from 178 and 168 ms during intrinsic rhythm to 120 and 128 ms during LBBP, respectively. In addition, one patient with RBBB exhibited lower QRS duration, from 188 to 130 ms by LBBP. Notably, the researchers found neither significant differences between the pacing thresholds (0.73 ± 0.20 V vs. 0.61 ± 0.23 V) nor adverse events during 3-month follow-up (18).

### Application of LBBP in CRT

Hou et al. (45) compared cardiac synchrony of LBBP with RVAP and HBP in bradycardia patients and found that QRS duration of LBBP was located between the other two (HBP vs. LBBP vs. RVSP; 99.7 ± 15.6 ms vs. 117.8 ± 11.0 ms vs. 158.1 ± 11.1 ms, *P* < 0.0001). Their results further revealed that LBBP patients with recorded LBB potential had the similar phase standard deviation (PSD) and phase histogram bandwidth (PHB) to those with HBP patients (PSD, 15.1° ± 5.3° vs. 13.9° ± 5.8°, *P* = 0.80; PHB, 46.2° ± 13.4° vs. 41.3° ± 12.6°, *P* = 0.51). In addition, LBBP resulted in lower pacing threshold (0.5 ± 0.1 V vs. 1.4 ± 0.8 V, *P* < 0.0001) and higher R-wave amplitude (17.0 ± 6.7 mV vs. 4.4 ± 4.3 mV, *P* < 0.0001) (45). Furthermore, Zhang et al. (20) performed LBBP in 11 HF patients with LBBB. Their results revealed significant narrowing of QRS duration following LBBP (139.09 ± 17.44 ms vs. 180.00 ± 15.86 ms), whereas the pacing threshold was low and stable. Moreover, all 11 patients exhibited a 5% improvement in their LVEF, relative to the baseline value, whereas seven of them had a 20% increase in LVEF and a 15% decrease in left ventricular end-systolic diameter (LVESD), respectively, relative to the baseline value (20). Wu et al. (21) reported the gratifying outcomes of CRT with LBBP in a non-randomized treatment comparison with HBP and BVP. Specifically, they analyzed a total of 137 patients with LVEF ≤ 40% and typical LBBB referred for CRT who received BVP, HBP, or LBBP and found mean paced QRS durations of 100.7 ± 15.3, 110.8 ± 11.1, and 135.4 ± 20.2 ms, respectively. Meanwhile, patients in the LBBP group had higher R-wave amplitude (11.2 ± 5.1 vs. 3.8 ± 1.9 mV) and lower pacing thresholds (0.49 ± 0.13 V/0.5 ms vs. 1.35 ± 0.73 V/0.5 ms) relative to those in the HBP group. Generally, both HBP and LBBP groups exhibited a similar absolute increase (Δ) in LVEF (+23.9 vs. +24%) and rate of normalized final LVEF (74.4 vs. 70.0%) at 1-year follow-up, which was significantly higher than those observed in the BVP group (ΔLVEF +16.7 and 44.9% rate of normalized final LVEF) (21). Moreover, Ravi et al. (47) reported that LBBP could significantly improve the left ventricular dysfunction in patients with HF during 6-month follow-up. Their results revealed significant improvement of LVEF (from 30 ± 11% to 42 ± 15%) following LBBP in 21 patients with cardiomyopathy. Among seven patients with LBBB and cardiomyopathy, the LVEF improved from 27 ± 4% to 36 ± 11%. In addition, there was a significant reduction in QRS duration (30–46 ms) in patients with baseline QRS duration > 120 ms. Recently, Huang et al. (48) also demonstrated that LBBP was a feasible and effective method for achieving electric resynchronization in patients with LBBB and non-ischemic cardiomyopathy in a prospective, multicenter study. Specifically, they recorded 97% (61/63) LBBP implantation success rates, with stable pacing threshold and R-wave amplitude at 1-year follow-up compared with implantation values (0.5 ± 0.15 V/0.5 ms vs. 0.58 ± 0.14 V/0.5 ms and 11.1 ± 4.9 mV vs. 13.3 ± 5.3 mV, respectively). Notably, the QRS duration narrowed from 169 ± 16 to 118 ± 12 ms during LBBP. In addition, patients exhibited a significant improvement in their LVEF (33 ± 8% vs. 55 ± 10%, *P* < 0.001) and a decrease in left ventricular end-systolic volume (123 ± 61 ml vs. 67 ± 39 ml, *P* < 0.001), relative to the baseline value (48).

## Advantages of LBBP

### RVAP and LBBP

Clinical practice has associated previous cardiac pacing strategies with deficiencies (49). RVAP is clearly non-physiological with regard to ventricular activation, with the creation of a LBBB-like sactivation sequence, and is associated with the risk of HF and AF as well as all-cause mortality (50). Alternative RV pacing sites, such as the right septum and right ventricular outflow tracts, have been attempted in the right ventricle, while their clinical outcomes remain controversial (5). Some studies have showed that LBBP confers better electrical and mechanical synchrony with RVAP and comparable R-wave amplitude and pacing threshold (18, 45), and its operation is safe and with few serious complications (18–20, 45). However, it is not known whether this approach's long-term clinical outcomes are superior to these of RVAP.

### HBP and LBBP

Theoretically, HBP is an ideal method for ventricular stimulation through the His–Purkinje conduction system. Numerous studies have demonstrated HBP's clinical benefits relative to those from RVAP in patients with preservation of LVEF (12). For example, permanent HBP has been proposed as an alternative to BVP for CRT (13). However, the His bundle is only ~1–2 mm in diameter, while HBP technique remains challenging (51). The His bundle is located in the central fibrous body and is minimally surrounded by myocardial tissue, which generates a high His capture threshold that may progressively increase during follow-up. Studies have also shown that HBP guarantees a higher 5-year generator replacement rate than RVAP (9 vs. 1%) (52). Capture thresholds required to correct underlying BBB are often higher in patients undergoing CRT with HBP, and their early battery depletion can still be a major obstacle (11). The mechanism through which HBP reverses LBBB is based on the concept of longitudinal dissociation with specific fibers within the His bundle committed to the left bundle. Thus, local lesions within the His bundle can result in LBBB, although this condition can be overcome by pacing at a location near or distal to the His bundle (53). Previous studies have shown that the mechanisms of LBBB are not restricted to the longitudinal dissociation of His. For instance, Upadhyay et al. (54) studied 85 patients with LBBB. They found that the cause of LBBB in 64% of cohort was localized conduction block, with no specific block but intraventricular conduction delay (IVCD) with intact Purkinje activation (IPA) in the remainder of the cohort. Patients with conduction block exhibited blockade, either at the level of the His bundle at the left septum (72%) or proximally within the left bundle (28%). Moreover, a majority of the patients with His block (94%) responded to HBP, compared to 64% of those with block in left bundle and none of the patients with IPA (54), indicating that LBBB may not be corrected by permanent HBP in 10–30% of patients (51). Notably, LBBP can bypass the pathological or disease-vulnerable region in the cardiac conduction system to produce near physiological or true conduction system pacing. In addition, a comparison with HBP indicates that LBBP operation is simple. Particularly, the entire LBB distribution area is similar to a “fan plane,” while the His bundle distribution is more restricted, in a similar fashion to a “point” (23). In fact, LBBP implantation guarantees a high success rate, between 80 and 97% (19, 34). Clinical studies have demonstrated that LBBP preserves better electrical and mechanical synchrony than RVAP, in a similar fashion to HBP. LBBP's R-wave amplitude and pacing threshold are reportedly more satisfactory and stable than those obtained in HBP (45). Furthermore, pacing at the LBB may also prevent later deterioration at the proximal His bundle or AV node, which may be caused by progression of AV conduction delay, and also provide more space for AV node ablation (17).

### BVP and LBBP

Currently, the application of BVP is the most common way of reversing or preventing pacing-induced dyssynchrony. Improvements in clinical applications have predisposed CRT to various shortcomings, with about 30% of patients reportedly not responding to the therapy (55, 56). Another problem associated with CRT via BVP is the use of epicardial LV pacing, which reverses physiologic activation of the ventricular wall. Functionally, this change increases transmural dispersion of repolarization (TDR) and QT interval, thereby creating a substrate for the development of torsade de pointe (TdP) (57). To date, the role of CRT in patients with preserved LV systolic function has not been elucidated. In addition, intravenous CRT implants are challenging, and diverse coronary sinus anatomy provides a limited choice of LV pacing sites. Consequently, research efforts have been directed to LV endocardial pacing. Mills et al. (58) demonstrated the benefits of LV endocardial pacing relative to traditional BVP, both acutely and chronically, and found that LV septal or apical pacing resulted in cardiac efficiency similar to that seen with native conduction. Other clinical studies have also demonstrated that LV pacing produces equivalent or even superior effects than conventional CRT via BVP (59, 60). However, LV endocardial pacing, via percutaneous atrial transseptal route, is complex and can influence mitral valve function and predispose patients to infections and stroke (61). Betts et al. (62) and Mafi-Rad et al. (63) reported a new feasible and safe route of LV endocardial pacing via ventricular septal puncture. Although LBBP can also be operated via transvenous approach through the interventricular septum, some differences have been reported between LVSP and LBBP. For instance, LBBP's lead position was higher than that of LVSP. The LBB potential, recorded in LBBP, shows that the pacing site is close to its torso and the Purkinje network, and this has not been reported in LVSP. In fact, LBBP's mean pacing QRS duration is narrower than the LVSP's, indicating the former's superiority with regard to ventricular synchrony (18, 19, 32, 45, 63). Apart from this, Li et al. (34) found that mechanical dispersion seemed to worsen over a 3-month follow-up period in three patients who received LVSP. However, LBBP could correct ventricular dyssynchrony, shorten QRS duration, promote LV reverse remodeling, and improve clinical symptoms in patients with HF (20, 21, 48).

## What Is the Future of LBBP Therapy?

Although early studies have demonstrated LBBP's potential as a physiologic pacing modality with stable and low threshold, numerous aspects of this therapy remain unknown, necessitating future explorations. For instance, what is the long-term safety and efficacy of the procedure? How can we accurately determine the depth of lead implantation to avoid the occurrence of interventricular septal perforation? Will the risk of thromboembolism and lead dislodgement increase? What is the long-term effect on interventricular septum and LBB when they are traumatized by the screw on the tip of the lead? Can a second LBBP lead be successfully placed if the earlier one fails in the long run? Beyond pacing hemodynamics, what is the impact of LBBP on arrhythmia? Since LBBP is also considered as a potential alternative to CRT, which patients with heart failure are best suited for LBBP, compared with either HBP or BVP? Apart from these areas, considerable efforts need to be directed to improving the design and structure of the lead as well as the delivery tools that will allow easier implantation and stabilization of the lead. Despite the technique's great potential for physiological pacing, further validation using studies with large numbers of participants and longer follow-up periods is required.

## Conclusions

Left bundle branch pacing is a novel pacing modality that can bypass the pathological or disease-vulnerable region in the cardiac conduction system, to provide physiological pacing modality for patients. LBBP guarantees a narrow paced QRS complex and fast LVAT, with a low pacing capture threshold. Previous studies have shown that LBBP can be applied to circumvent the limitations of HBP or RV pacing and can acts as a potential alternative to CRT in patients with typical LBBB. Future studies are expected to validate LBBP's safety, reliability, and long-term performance using large prospective trials and affirm its potential as an alternative option for physiological pacing in several groups of patients.

## Author Contributions

QZ and XQ provided the idea and technical guidance for the manuscript. PL wrote the manuscript. QW and HS made the figures. All authors have read and agreed to the published version of the manuscript.

## Conflict of Interest

The authors declare that the research was conducted in the absence of any commercial or financial relationships that could be construed as a potential conflict of interest.
